# In Vitro Co-Exposure to CeO_2_ Nanomaterials from Diesel Engine Exhaust and Benzo(*a*)Pyrene Induces Additive DNA Damage in Sperm and Cumulus Cells but Not in Oocytes

**DOI:** 10.3390/nano11020478

**Published:** 2021-02-13

**Authors:** Martina Cotena, Mélanie Auffan, Virginie Tassistro, Noémie Resseguier, Jérôme Rose, Jeanne Perrin

**Affiliations:** 1 IMBE, CNRS, IRD, Avignon Université, Aix Marseille Univ., 13005 Marseille, France; martina.cotena@univ-amu.fr (M.C.); virginie.tassistro@univ-amu.fr (V.T.); 2 CEREGE, CNRS, Aix Marseille Univ., IRD, INRAE, Coll France, 13545 Aix-en-Provence, France; auffan@cerege.fr (M.A.); rose@cerege.fr (J.R.); 3 Civil and Environmental Engineering, Duke University, Durham, NC 27708, USA; 4 Department of Biostatistics and Public Health, La Timone Hospital, 13005 Marseille, France; noemie.resseguier@univ-amu.fr; 5 Laboratory of Reproduction Biology-CECOS, Department of Gynecology, Obstetrics and Reproductive Medicine, AP-HM La Conception, Pôle Femmes Parents Enfants, 13005 Marseille, France

**Keywords:** genotoxicity, nanomaterials, polycyclic aromatic hydrocarbons, germ cells, additivity, cocktail

## Abstract

Benzo(a)pyrene (B*a*P) is a recognized reprotoxic compound and the most widely investigated polycyclic aromatic hydrocarbon in ambient air; it is widespread by the incomplete combustion of fossil fuels along with cerium dioxide nanomaterials (CeO_2_ NMs), which are used in nano-based diesel additives to decrease the emission of toxic compounds and to increase fuel economy. The toxicity of CeO_2_ NMs on reproductive organs and cells has also been shown. However, the effect of the combined interactions of B*a*P and CeO_2_ NMs on reproduction has not been investigated. Herein, human and rat gametes were exposed in vitro to combusted CeO_2_ NMs or B*a*P or CeO_2_ NMs and B*a*P in combination. CeO_2_ NMs were burned at 850 °C prior to mimicking their release after combustion in a diesel engine. We demonstrated significantly higher amounts of DNA damage after exposure to combusted CeO_2_ NMs (1 µg·L^−1^) or B*a*P (1.13 µmol·L^−1^) in all cell types considered compared to unexposed cells. Co-exposure to the CeO_2_ NMs-B*a*P mixture induced additive DNA damage in sperm and cumulus cells, whereas no additive effect was observed in rat oocytes. This result could be related to the structural protection of the oocyte by cumulus cells and to the oocyte’s efficient system to repair DNA damage compared to that of cumulus and sperm cells.

## 1. Introduction

Diesel engines are one of many sources of ambient particulate matter and gaseous air pollutants [1]. Diesel exhaust is a complex mixture of particles, commonly known as soot and gases and contains more than one hundred different organic and inorganic compounds, including many chemicals that have been designated as air pollutants [2]. In 2012, the International Agency for Research on Cancer (IARC), part of the World Health Organization (WHO), upgraded the carcinogenicity of diesel emissions from Group 2 A (probably carcinogenic) to Group 1 (carcinogenic with sufficient evidence) [3]. For instance, diesel engines are significant sources of polycyclic aromatic hydrocarbons (PAHs) in urban air [4]. Despite the hazards induced by PAHs to humans, there are no motor vehicle emission limits for these compounds in most countries. Sixteen PAHs compounds have been classified by the U.S. EPA as a priority pollutant because of various toxicological concerns [5] and significant health impacts [6]. Among them, benzo*a*pyrene (B*a*P) is recognized as a powerful carcinogen, mutagen, and reprotoxic compound [7,8]. The exposure to PAHs is mostly through ingestion and air inhalation, the B*a*P “virtually safe dose” is depending on countries legislation and is between 0.7–1 ng/m^3^ [9]. B*a*P is associated with increased genotoxicity [10,11,12] and DNA fragmentation [13] towards sperm cells and oocytes. B*a*P exposure decreases sperm motility and morphology and increases DNA damage [14,15,16]. In vivo experimental studies have also shown that postnatal exposure to B*a*P destroys ovarian follicles due to the inhibition of follicle growth and then causes premature ovarian failure [17,18,19,20]. More recently, nanomaterials (NMs) have been increasingly used in Europe and elsewhere as fuel-borne catalysts in diesel engines [21,22,23] as CeO_2_ NMs [24,25]. These CeO_2_ NMs are used to decrease the emission of toxic compounds in exhaust [26], but they have also been shown to increase the emission of ultrafine particles and the amount of Ce released [26]. Compared to that of B*a*P, the potential effect of the released CeO_2_ NMs on health is still not fully understood [27,28], and up to now, there are still few studies regarding the exposure to CeO_2_ NMs, and no secure data are reported concerning the humans exposure limits. However, few in vivo and in vitro studies have demonstrated the potential toxicity of CeO_2_ NMs on reproductive cells [29,30,31,32,33,34], which likely occurs via the generation of reactive oxygen species (ROS), leading to oxidative stress and DNA damage [26,32,33]. Interestingly, the biological effects of NMs depend not only on their own structure and chemistry but also on their interactions (e.g., adsorption, complexation) with other pollutants, such as PAHs, metals, metalloids, etc. [35,36]. To date, most research on the effects of chemicals on biological systems is conducted on one chemical at a time, while in the real world (as with diesel exhaust), people are exposed to chemical mixtures whose effects are extremely complex and need further investigation [37]. Within mixtures, chemicals (organic, inorganic, dissolved, and nanoparticulate) could interact additively (which results in the sum of toxicity of each agent), synergistically (inducing toxic effects greater than the sum of the effects of the individual chemicals) or antagonistically (where the combined effect of two or more compounds is less toxic than the individual effects) [38]. This study aimed to investigate the combined biological effects of one commercialized CeO_2_ NM-based diesel additive (Envirox^TM^ from Energenics Europe Ltd., Begbroke, UK) and one PAH (B*a*P), both of which are likely released in the atmosphere after combustion in a diesel engine [4,25,39]. Prior to the in vitro exposure of germ cells, Envirox^TM^ was combusted at 850 °C to mimic its physico-chemical transformations in a diesel engine [40]. Then, the potential genotoxicity induced by the in vitro co-exposure of human and rat gametes to combusted CeO_2_ NMs along with B*a*P was investigated using the comet assay. Herein, we will study how the interactions between combusted CeO_2_ NMs and B*a*P molecules in diesel exhaust may additively, synergistically, or antagonistically impact the previously observed genotoxicities of the individual compounds on human and rat germ cells (sperm, follicular cells, and oocytes).

## 2. Materials and Methods

### 2.1. Solution and Suspension Preparation Prior to Exposure

Metabolic activation of benzo(a)pyrene (B*a*P*)*. B*a*P was purchased from Sigma Aldrich (Saint-Quentin Fallavier, France). A B*a*P stock suspension was prepared in dimethyl sulfoxide (DMSO) (Sigma Aldrich) at 10 mM to obtain complete dissolution [41]. To activate B*a*P metabolism, we used an S9 mix [39,42,43] that consisted of the following cofactors: pooled S9 rat liver (Sigma Aldrich), 1 M KCl, 0.25 M MgCl_2_*6H_2_O, 0.2 M glucose-6-phosphate, and 0.04 M NADP [44]. The final concentration of B*a*P at 1.13 µmol·L^−1^ was then prepared in Ferticult^®^ medium (JCD Laboratories, Lyon, France), with 1% S9 mix and 1% DMSO as previously described by Baumgartner et al. (2012) [45]. The working concentration was mainly chosen because of previously published toxicological data, but also due to the solubility limits in biological media [45].

Aging of the diesel fuel additive. CeO_2_ NMs were extracted from Envirox^TM^, a fuel-borne catalyst scientifically and commercially proven CeO_2_ NM-based diesel additive supplied by Energenics Europe Ltd. The Envirox^TM^ was combusted and characterized following the protocol already published in ref [40,46]. Briefly, the Envirox^TM^ was by ultracentrifugated at 396,750x g and 20 °C for 1 h. The pellets containing CeO_2_ NMs were freeze-dried (Heto PowerDry LL3000, Thermo Fisher Scientific, Strasbourg, France) for 5 days and combusted at 850 °C [30,40]. A stock suspension of the combusted Envirox^TM^ (called aged CeO_2_ NMs) was prepared in Milli-Q water at 10.15 g·L^−1^ CeO_2_ and put under magnetic stirring to avoid the formation of large aggregates. The final concentration (1 µg·L^−1^) was prepared in Ferticult^®^ medium. This concentration of CeO_2_ NMs was chosen because it was the lowest studied concentration responsible for significant DNA damage in human and rat sperm cells [30].

Mixture of aged CeO_2_ NMs and B*a*P. One µg·L^−1^ of aged CeO_2_ NMs was incubated with 1.13 µmol·L^−1^ B*a*P in abiotic Ferticult^®^ supplemented with 1% S9 mix and 1% DMSO for 1 h at room temperature (RT) prior to exposure to the cells. To estimate the stability of B*a*P in supplemented Ferticult^®^, pure suspensions of B*a*P at 50 µmol·L^−1^ were also incubated without NMs in supplemented Ferticult^®^, centrifuged (1 h at 4000× *g*), or settled (1 h), and their supernatant was measured by UV-vis spectrometry (mySPEC Twin UV-vis spectrometer, VWR, Val-de-Marne, France). Standard curves obtained at two wavelengths corresponding to the B*a*P signal (300 and 384 nm) are provided in Supporting Information. We estimated that 30 ± 6% of the B*a*P was removed from the solution just by 1h settling and 57 ± 11% by 1h centrifugation. This could highlight the incomplete dissolution but also to the chemical instability of B*a*P in these abiotic conditions related to its high affinity for serum components (i.e., as albumin in Ferticult^®^) [47,48,49,50]. UV-vis spectrometry was used to estimate the affinity of B*a*P for the surface of the aged CeO_2_ NMs in abiotic conditions. To be in the detection range of the apparatus (see standard curves in Supplementary Materials, Figure S1), 10 µg·L^−1^ aged CeO_2_ NMs were mixed with 11.3 µmol·L^−1^ B*a*P (similar [CeO_2_]/[B*a*P] ratio of concentration to those used with the cells) in Ferticult^®^ medium supplemented with 1% S9 mix and 1% DMSO for 1 h under mechanical stirring at RT. After 1 h, the samples were centrifuged (1 h at 4000× *g*), and the supernatant was recovered. No washing step was performed in order to access both the weak and strong surface affinity of B*a*P for NMs. The absorbance corresponding to B*a*P was measured in the supernatant by UV-vis at two wavelengths (300 and 384 nm). The percentage of B*a*P adsorbed at the surface of NMs was estimated taking into account the B*a*P instability in abiotic Ferticult^®^ (with NMs) following centrifugation.

### 2.2. Gamete Collection

Rat cumulus–oocytes complex (COC) collection. Female superovulation was induced in prepubescent rats by an intraperitoneal injection of pregnant mare serum gonadotropin (20 U.I. PMSG) on day one and human chorionic gonadotropin (40 U.I. HCG) on day three. Twelve hours later, we collected oviducts containing oocytes surrounded by follicle cells after cervical dislocation euthanasia [51]. Once the cells from each oviduct were recovered, we left them equilibrate in Ferticult^®^ medium at 37 °C and CO_2_ 5% for 1 h [46].

Rat sperm cell collection. Male rats were previously anesthetized (Sevoflurane, vol % 8) and then euthanized with a 10 mL injection of Dolethal. After sacrifice, we collected and cut the epididymis to allow the exit of sperm into HTF-BSA culture medium (Human Tubal Fluid, Millipore, St-Quentin-en-Yvelines, France, with 0.4% BSA: Bovine Serum Albumin, Sigma-Aldrich, St. Quentin-Fallavier, France) for 1 h at 37 °C and CO_2_ 5% under mineral oil (Sigma-Aldrich^®^, France) [30].

Human sperm collection. We used frozen human sperm from healthy fertile donors. After thawing, we aliquoted the preparation and centrifuged it for 10 minutes at 420× *g*. The supernatants were discarded, and the pellets were exposed to various exposure conditions [30].

### 2.3. Ethical Authorization

Ethical authorization for animal sampling of gametes was obtained from the National Ethics Committee on Animal Experimentation (2018061110211950-V2 #15447). We used Sprague-Dawley rats, Oncins France Strain A (623OFA), which were purchased from Charles River Laboratories (Lyon, France). Sexually mature 60-day-old male rats and prepubescent 26-day-old female rats were housed with free access to food and water until sacrifice.

Human sperm cells were purchased from GERMETHEQUE Biobank (BB-0033-00081 Marseille, France); informed consent was obtained from each donor for the inclusion of samples in the biobank and for their use in research experiments regarding human fertility in accordance with the 1975 Helsinki Declaration on human experimentation. The Scientific Committee approved the present study design (number 20130102).

### 2.4. Gamete Exposure and DNA Damage Evaluation by the Comet Assay

We exposed human sperm, rat sperm, and COCs to three experimental conditions: (i) aged CeO_2_ NMs at 1 µg·L^−1^ (called NMs); (ii) B*a*P at 1.13 µmol·L^−1^ (called B*a*P); (iii) aged CeO_2_ NMs at 1 µg·L^−1^ previously incubated with 1.13 µmol·L^−1^ BaP (called NMs+B*a*P). FertiCult^®^ medium alone and Ferticult^®^ medium containing 1% S9 mix and 1% DMSO were used as the negative control and internal control (IC), respectively. As a protocol verification, we also exposed rat sperm cells to Ferticult^®^ medium 1% S9 mix, 1% DMSO, and CeO_2_ NMs (1 µg·L^−1^) (see Supplementary Materials, Figure S2). H_2_O_2_ (110 µmol·L^−1^) in Ferticult^®^ medium was used as a positive control, and the H_2_O_2_ concentration was chosen based on previous studies [11,31,32]. At least three different experiments were performed for each condition. After exposure, we recovered all motile sperm cells by swim-up [8], and we measured sperm viability by eosin-nigrosine staining according to the WHO (WHO, 1999, Appendix IV.2) technique (100 cells were evaluated per condition). We then performed the alkaline comet assay according to the procedure described by Singh et al. (1988) [52] and adapted by Baumgartner et al. (2009) [53], which has already been described in ref [30,31]. DNA damage was quantified by the percentage of DNA in the tail of 100 randomly selected sperm cells from each triplicate slide per condition (at least 300 raw values analyzed per experiment, at least 900 in total per condition). Regarding the COC, we performed a comet assay according to the protocol described by Berthelot-Ricou et al. (2011) [54] and adapted by Préaubert et al. (2015) [32]. DNA damage was quantified by Olive Tail Moment (OTM) [55] in 2 replicated slides of each condition per experiment (at least 100 cumulus cells per experiment, 300 in total per condition, and at least 30 oocytes per experiment, 90 in total per condition).

The data are presented as the medians of % tail DNA or olive tail moment (OTM) values with 1st and 3rd quartiles. We performed a linear mixed model analysis with “condition” (exposure condition) as a fixed effect and “cells” (sperm cells, follicle cells, or oocytes) within the replicate slide as a random effect using the linear mixed effects regression (LMER) function of R software, version 3.6.0 (R Foundation for Statistical Computing, Vienna, Austria), to compare DNA damage among the various conditions. Pairwise differences of least-square means for all conditions were post hoc assessed. Statistical significance was set at *p* < 0.05.

## 3. Results and Discussion

### 3.1. DNA Damage in Sperm Cells Induced by Aged CeO_2_ NMs and/or BaP

**Figure 1:**
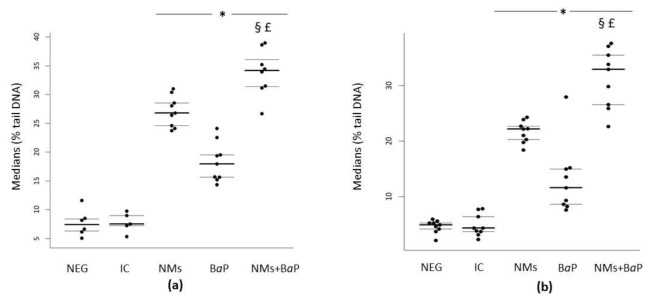
Evaluation of DNA damage using the comet assay following in vitro exposure of human (**a**) and rat sperm (**b**) to NMs+B*a*P. Tested concentrations: Negative control = Figure 1. S9 mix, 1% DMSO), NMs: aged CeO_2_ NMs at 1 µg·L^−1^; B*a*P: B*a*P at 1.13 µmol·L^−1^; NMs+B*a*P: aged CeO_2_ NMs at 1 µg·L^−1^ previously incubated with 1.13 µmol·L^−1^ B*a*P. *p* < 0.05, for differences compared versus *: negative control (NEG); §: vs. NMs, £: vs. B*a*P.

In human and rat sperm cells, a significant increase in DNA damage was observed after 1 h of in vitro exposure to NMs+B*a*P versus that in the negative control and NMs and B*a*P alone groups (*p* < 0.001) (Figure 1a,b, Table 1). It is noteworthy that all the viability rates were over the normality threshold as stated by the WHO criteria [56]. The results are presented as the distribution of median values of the % tail DNA with 1st and 3rd quartiles obtained from three independent experiments. These values could inform about additive, synergistic, or antagonistic effects within the mixture [57]. In both human and rat sperm cells, a significantly higher genotoxicity was detected after exposure to the NM+B*a*P mixture compared to the toxicity of single contaminants, highlighting the additive effects of NMs and B*a*P when sperm cells are simultaneously exposed.

NMs and B*a*P are known to individually induce DNA damage on sperm cells, resulting in adverse effects on the fertilization rate [32] and sperm nucleus [8]. Our previous in vitro studies showed a significant increase in DNA damage in human sperm after exposure to 10 µg·L^−1^ of pristine CeO_2_ NMs. The mechanisms of the genotoxicity were indirectly attributed to oxidative stress via the adjunction of an antioxidant (L-ergothioneine) in the exposure medium [31]. We also observed a significant increase in intracellular ROS production after in vitro exposure to 1 µg·L^−1^ of aged CeO_2_ NMs in human sperm cells. This enhanced oxidative stress was attributed to a potential reductive dissolution of Ce(IV) in the vicinity of the plasma membrane of the cells into Ce(III) with pro-oxidant abilities [30]. It is noteworthy that CeO_2_ NM internalization within sperm cells was never observed under any exposure condition [30,31].

Conversely, it is well known that B*a*P directly penetrates sperm cells. Its metabolism involves the activation of the aryl hydrocarbon receptor, which increases the expression of cytochrome P450 1A1 and 1B1, followed by the generation of reactive metabolites (4,5-diol, 7,8-diol, and 9,10-diol). After the reactive bay region, diol epoxide may covalently bind to DNA and other cellular macromolecules, which initiate its toxicity, mutagenesis, and carcinogenesis [58]. B*a*P exposure in human is associated with BPDE-DNA adducts and ROS production in sperm [8,20,59,60,61,62]. Moreover, Zhang et al. (2019) recently demonstrated that in vivo exposure to B*a*P can also significantly change the DNA methylation of rat sperm, mainly through hypomethylation [63]. These changes are associated with alterations in embryonic and reproductive system development and with many genetic diseases, but it is still not understood whether these epigenetic changes are transgenerational and can then be transmitted to offspring [63].

Few recent toxicological studies have started considering the co-exposure to NMs and other contaminants [57]. For instance, Asweto et al. (2017) showed for the first time a synergistic interaction between Si-based NMs and B*a*P involved in enhancing their individual toxicity after in vitro co-exposure of endothelial cells. It causes excessive oxidative stress, leading to DNA damage, cell cycle arrest, and apoptosis [35]. Herein, we assessed whether the physicochemical interactions between B*a*P and aged CeO_2_ NMs might modify the behavior of B*a*P under abiotic conditions using UV-vis spectrometry. For [CeO_2_] over a [B*a*P] ratio of concentrations similar to that used with the cells (11.3 µmol·L^−1^ B*a*P for 10 µg·L^−1^ CeO_2_ NMs), we estimated that 44 ± 9% of B*a*P interacted with the surface of the NMs. This affinity is in agreement with previous studies showing the effect of ultrafine, airborne, carrier (nano)particles on the deposition, retention, and biological fate of PAHs [64,65,66,67]. Herein, we demonstrated that co-exposure to aged CeO_2_ NMs and B*a*P additively impact sperm cells. Consequently, the potential affinity of the B*a*P for the CeO_2_ NMs surface observed in abiotic media did not impact the toxicity. This could be either attributed to the B*a*P desorption from the CeO_2_ NMs surface related to reductive dissolution of nanocrystalline Ce(IV)O_2_ into Ce(III) at the vicinity of the cell membrane [30], but also to the limited number of B*a*P binding sites at the surface of the sperm cells and to the limited capacity of the constitutive CYP1A (cytochrome P4501A) enzymatic activity in sperm [47].

### 3.2. DNA Damage in COCs Induced by Aged CeO_2_ NMs and/or BaP

Interactions and close communication between cumulus cells and oocytes in COCs are critically important for oocyte maturation and quality. Cumulus cells are particularly sensitive to exogenous contaminants [68] and provide oocyte protection against short-lived perturbations in the surrounding environment [69,70,71].

**Figure 2:**
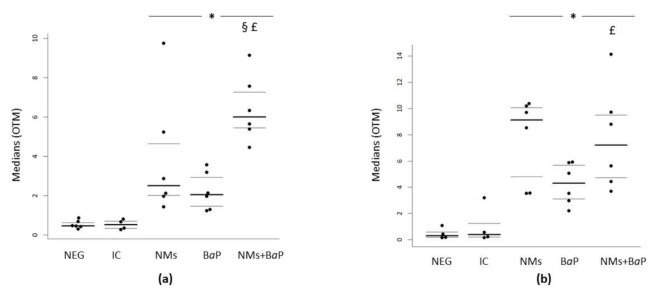
Evaluation of DNA damage using the comet assay following in vitro exposure of rat cumulus cells (**a**) and oocytes (**b**) to NMs+B*a*P. Tested concentrations: negative control = Ferticult^®^ medium, IC = intern control (Ferticult^®^ 1% S9 mix, 1% DMSO), NMs: aged CeO_2_ NMs at 1 µg·L^−1^; B*a*P: B*a*P at 1.13 µmol·L^−1^; NMs+B*a*P: aged CeO_2_ NMs at 1 µg·L^−1^ previously incubated with 1.13 µmol·L^−1^ B*a*P. *p* < 0.05, for differences compared versus *: negative control (NEG); §: vs. NMs, £: vs. B*a*P.

In rat cumulus cells, significantly higher DNA damage was observed after 1 h of in vitro exposure to the NMs+B*a*P mixture compared to the negative control, NMs alone, and B*a*P alone (*p* < 0.001) (Figure 2a and Table 2). The results are presented as the distribution of median values of OTM with 1st and 3rd quartiles obtained from three independent experiments. The significantly different toxicities observed after exposure to the NM+B*a*P mixture highlight the additive effect of NMs and B*a*P upon co-exposure to CCs.

It is well known that B*a*P metabolites impair follicle growth in vitro and increase primordial follicle atresia through the induction of apoptosis [72,73,74,75]. Siddique et al. (2013) demonstrated that in vitro exposure to B*a*P [1.5–45 µg·L^−1^] for 13 days induces oxidative stress in cumulus cells, highlighted by a significant increase in 8-OH-dG, which is a general biomarker of cellular oxidative stress and DNA oxidative damage [76]. Einaudi et al. (2014) showed a significant increase in DNA damage and BPDE-DNA adducts in cumulus cells after in vivo exposure to a single dose of B*a*P [13 mg/kg body weight] [11]. They observed B*a*P-induced genotoxicity [11], which was related to the different follicle maturation stages [77,78,79]. Conversely, there is still a large gap in the literature regarding the potential effect induced by NMs exposure on cumulus cells. A few previous studies reported a significant dose-dependent genotoxicity in cumulus cells exposed in vitro to 2.10^3^ to 1·10^5^ µg·L^−1^ pristine NMs, likely related to oxidative stress [33]. Moreover, during in vitro exposure of COCs to NMs, Courbiere et al. (2013) showed the ability of cumulus cells to internalize pristine CeO_2_ NMs (~8 nm) by endosomal trapping after in vitro exposure to 10·10^4^ µg·L^−1^ CeO_2_ NMs [32,33]. Based on this internalization and contrary to the case of sperm cells, a so-called “Trojan horse effect” could have occurred in cumulus cells. Indeed, metal oxide NMs have already been shown to enhance the toxicity of contaminants adsorbed on their surface via modification of their bioavailability [57]. However, Figure 2a shows that despite the affinity of B*a*P for the surface of aged CeO_2_ NMs, co-exposure to NMs and B*a*P resulted in additive genotoxicity of the two single contaminants towards cumulus. Consequently, no Trojan horse effect modifying the toxicity of B*a*P or aged CeO_2_ NMs has been observed under our experimental conditions.

In rat oocytes, we detected a significant increase in DNA damage after in vitro exposure to NMs+B*a*P compared to the negative control and B*a*P alone (*p* < 0.001). In contrast to sperm and cumulus cells, we did not observe any significant difference in NMs+B*a*P exposure versus NMs alone (*p* > 0.05) (Figure 2b, Table 2). This result did not highlight any additive effect when oocytes were co-exposed to NMs and B*a*P. The results are presented as the distribution of median values of OTM with 1st and 3rd quartiles obtained from three independent experiments.

Few studies have explored the effect of CeO_2_ NMs on oocytes. In mouse the genotoxicity induced at 10 µg·L^−1^ of pristine CeO_2_ NM was attributed to oxidative stress [32]. Despite the protection of the zona pellucida, TEM analysis showed pristine CeO_2_ NMs in the perivitelline space (between the plasma membrane and the zona pellucida) after in vitro exposure to 10·10^4^ µg·L^−1^ [32], highlighting the incomplete protection of cumulus cells against contaminants. Regarding B*a*P toxicity towards oocytes, it has been shown that the ovary possesses the ability to metabolically process B*a*P and obtain more reactive intermediates [80,81]. The generation of these metabolites is of importance, as they are capable of inducing cellular toxicity through the production of ROS and oxidative DNA damage [82], which has been linked to B*a*P-induced subfertility [83]. Rekhadevi et al. (2014) demonstrated that in vitro exposure of human ovarian subcellular fractions to 1 and 3 µmol·L^−1^ B*a*P induces metabolite accumulation, which contributes to premature ovarian failure [80]. An in vivo study showed that B*a*P oral exposure induced oxidative stress with an increased level of ROS and apoptosis in cumulus-denuded oocytes in mice after administration of B*a*P (10, 20, or 40 mg/kg body weight per day for 10 d), highlighting that oxidative stress is one of the mechanisms responsible for B*a*P metabolite-induced toxicity [84]. Additionally, Einaudi et al. (2014) also detected a significant increase in DNA damage in mouse oocytes after oral in vivo exposure to a single dose of B*a*P (13 mg/kg body weight) depending on the maturation stages [11]. The lower sensitivity of mature oocytes (exposed in antral follicles) to B*a*P-induced DNA damage could be due to oocytes that have reached the nuclear maturity required to repair DNA damage [77,78,79]. It has been shown that even the zona pellucida protects the oocytes, excluding some contaminants [85]; most biologically active molecules can pass through independently of the developmental stage [86].

Herein, we demonstrated that NMs+B*a*P exposure of oocytes did not induce any additive effect compared to NMs exposure alone, contrary to what we observed with sperm and cumulus cells. This result could be explained by the particular architecture and biology of COCs. First, there is structural protection around the oocyte due to the multiple layers of zona pellucida and cumulus cells [87,88]. These protective layers are gatekeepers for the oocyte [88,89] and act as a barrier between the oocyte and the extrafollicular environment [74,90,91], with cumulus cells able to select and process the metabolites that oocyte will receive [89]. This protection limits the contact between CeO_2_ NMs and the oocyte plasma membrane compared to CeO_2_ NMs interactions with sperm and cumulus cells. Second, in contrast to sperm and cumulus cells, oocytes have an efficient system to repair a variety of DNA lesions [92]. DNA repair activity in the zygote and during early development is, by definition, of maternal origin [93]. It is particularly important for germ cells to correct damage to their DNA, to avoid apoptosis, and prevent the transmission of genetic mutations to offspring [90,94]. Instead, sperm cells generally lack cytosolic antioxidants and fully functional DNA repair machinery, as they only possess the first enzyme in the base-excision repair pathway, OGG1, which removes the oxidized base, leaving a vulnerable abasic site [91]. Consequently, following the co-exposure of oocytes, efficient system repair protected the cells from NM-B*a*P-induced oxidative DNA damage, therefore resulting in a non-additive effect of the mixture of contaminants.

## 4. Conclusions

Drawing upon previous studies, we investigated the potential interaction between aged CeO_2_ NMs and B*a*P and the consequential impact on reproductive cells. We demonstrated additive toxic effects of NM+B*a*P exposure on sperm and cumulus cells compared to those generated by the individual pollutants. However, we did not show any additive effect in rat oocytes. This was attributed to the protection of the oocyte by the cumulus cells and to the oocyte’s efficient system to repair DNA damage compared to that of cumulus and sperm cells. The exposure of COCs and the subsequent genotoxic analysis by the comet assay of both cell types separately allowed us to analyze the impact of cumulus cells on the DNA damage of oocytes, which complies with the real exposure conditions. To further understand the impact of co-exposure on reproduction, in vivo studies are required. *In vivo*, the behavior, the time exposure, and fate of the two pollutants are expected to be different, which should affect their bioavailability, bioaccumulation, and toxicity.

## 5. Limitations and Strengths

Our study considers for the first time a co-contamination scenario of germ cells that is close to the real conditions in which humans are likely exposed to different emissions of ultrafine particles and many other pollutants. We considered the potential exposure of human and rat gametes to the combination of aged CeO_2_ NMs and B*a*P, which are released in the atmosphere after combustion in a diesel engine. The CeO_2_ NMs used in this study are representative of nano-based diesel fuel additives likely released by combustion in a diesel engine and to which people are potentially exposed [30]. Even though this study reflects realistic exposure conditions because of co-exposure to low concentrations of aged CeO_2_ NMs and B*a*P, it is limited by its in vitro nature.

## Figures and Tables

**Table 1:** Median values of the % tail DNA of each condition of three experiments, with 1st and 3rd quartiles, in rat and human sperm.

	Rat Sperm			Human Sperm		
Condition	MEDIAN Values	1st Quartile	3rd Quartile	MEDIAN Values	1st Quartile	3rd Quartile
Negative control	4.9	4.17	5.3	7.39	6.27	8.39
IC	4.34	3.73	6.4	7.49	7.20	8.99
NMs	22.15	20.3	22.68	26.78	24.62	28.55
B*a*P	11.64	8.63	14.99	17.94	15.64	19.53
NMs+B*a*P	32.88	26.57	35.44	34.19	31.4	36.06

**Table 2:** Median values of % olive tail moment (OTM) of each condition of three experiments, with 1° and 3° quartiles, in cumulus–oocytes complexes (COCs).

	Rat Cumulus Cells			Rat Oocytes		
Condition	MEDIAN Values	1st Quartile	3rd Quartile	MEDIAN Values	1st Quartile	3rd Quartile
Negative control	0.46	0.43	0.62	0.3	0.17	0.58
IC	0.51	0.33	0.70	0.39	0.20	1.23
NMs	2.49	2	4.64	9.12	4.8	10.07
B*a*P	2.04	1.46	2.92	4.3	3.12	5.68
NMs+B*a*P	5.99	5.44	7.26	7.22	4.74	9.5

## Data Availability

Data is contained within the article or supplementary material.

## Supplementary Materials

The following are available online at https://www.mdpi.com/2079-4991/11/2/478/s1, Figure S1. Supplementary controls used during the assessment of DNA damage by the comet assay. Figure S2. Standard curves for BaP measurements by UV-Vis spectrometry.
