# Supplementary material for: In Vitro Co-Exposure to CeO2 Nanomaterials from Diesel Engine Exhaust and Benzo(a)Pyrene Induces Additive DNA Damage in Sperm and Cumulus Cells but Not in Oocytes
Source: Nanomaterials (Basel). 2021 Feb 13;11(2):478. doi: 10.3390/nano11020478 (PMC7918929; doi:10.3390/nano11020478)
Supplement: Supplementary file 1 [file nanomaterials-11-00478-s001.zip › nanomaterials-1101182-supplementary.docx]

**SUPPORTING INFORMATION**

*In Vitro* Co-Exposure to CeO_2_ Nanomaterials from Diesel Engine Exhaust and Benzo(*a*)Pyrene Induces Additive DNA Damage in Sperm and Cumulus Cells but not in Oocytes

**Martina Cotena ^1,2^, Mélanie Auﬀan ^2,3^, Virginie Tassistro ^1^, Noémie Resseguier ^5^, Jérôme Rose ^2,3^ and Jeanne Perrin ^1,6*^**

^1^ IMBE, CNRS, IRD, Avignon Université, Aix Marseille Univ, 13005 Marseille, France; martina.cotena@univ-amu.fr (M.C.); virginie.tassistro@univ-amu.fr (V.T.)

^2^ CEREGE, CNRS, Aix Marseille Univ, IRD, INRAE, Coll France, Aix-en-Provence, France; auﬀan@cerege.fr (M.A.); rose@cerege.fr (J.R.)

^3^ Civil and Environmental Engineering, Duke University, Durham, NC 27708, USA

^4^ Department of Biostatistics and Public Health, La Timone Hospital, 13005 Marseille, France; noemie.res-seguier@univ-amu.fr

^5^ Laboratory of Reproduction Biology-CECOS, Department of Gynecology, Obstetrics and Reproductive Medicine, AP-HM La Conception, Pôle Femmes Parents Enfants, 13005 Marseille, France

***Correspondence**: [jeanne.perrin@univ-amu.fr](mailto:jeanne.perrin@univ-amu.fr)

List of Figures:

Figure S1. DNA damage assessed using the Comet Assay following *in vitro* exposure of rat sperm to aged CeO_2_ NMs in different media. Negative control: Ferticult® medium, IC: Intern control (Ferticult® 1% S9 mix, 1% DMSO), IC-NMs: Ferticult® 1% S9 mix, 1% DMSO, 1 µg.L^-1^ of aged CeO_2_ NMs. *p* <0.05, for differences compared *versus* *: negative control (NEG).

Figure. S2 Standard curves obtained at two wavelengths (300 and 384 nm) corresponding to the B*a*P signal by UV-Vis spectrometry analysis.


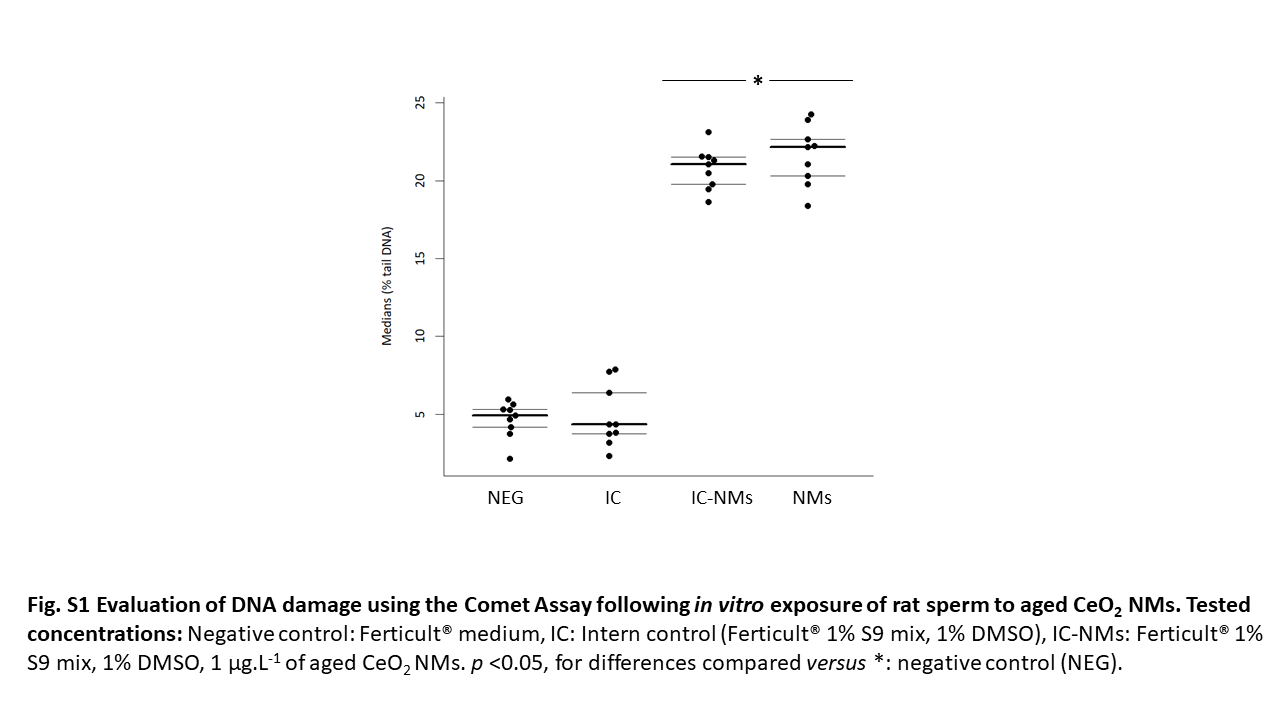


Figure S1. DNA damage assessed using the Comet Assay following *in vitro* exposure of rat sperm to aged CeO_2_ NMs in different media. Negative control: Ferticult® medium, IC: Intern control (Ferticult® 1% S9 mix, 1% DMSO), IC-NMs: Ferticult® 1% S9 mix, 1% DMSO, 1 µg.L^-1^ of aged CeO_2_ NMs. *p* <0.05, for differences compared *versus* *: negative control (NEG).

**Figure. S2** Standard curves obtained at two wavelengths (300 and 384 nm) corresponding to the B*a*P signal by UV-Vis spectrometry analysis.
